# Clinical impact of high-quality testing for peritoneal lavage cytology in pancreatic cancer

**DOI:** 10.1038/s41598-024-60936-4

**Published:** 2024-05-03

**Authors:** Masahiro Tanemura, Kenta Furukawa, Manabu Mikamori, Tadafumi Asaoka, Hironao Yasuoka, Daiki Marukawa, Yasuo Urata, Daisaku Yamada, Shogo Kobayashi, Hidetoshi Eguchi

**Affiliations:** 1 Department of Surgery, Rinku General Medical Center, 2-23 Rinku Orai-kita, Izumisano, Osaka 598-8577 Japan; 2 Department of Surgery, Osaka Police Hospital, 10-31 Kitayamachyo, Tennouji-ku, Osaka 543-0035 Japan; 3 Department of Pathology, Osaka Police Hospital, 10-31 Kitayamachyo, Tennouji-ku, Osaka 543-0035 Japan; 4 Oncolys BioPharma Inc., Toranomon Towers 10F, 4-1-28 Toranomon, Minato-ku, Tokyo 105-0001 Japan; 5 Department of Gastroenterological Surgery, Graduate School of Medicine and Faculty of Medicine, Osaka University, 2-2 Yamadaoka, Suita, Osaka 565-0871 Japan

**Keywords:** Pancreatic cancer, Peritoneal lavage cytology, Liquid biopsy, Peritoneal recurrence, Occult metastasis, Staging laparoscopy, Cancer, Biomarkers

## Abstract

In pancreatic ductal adenocarcinoma (PDAC) patients, the importance of peritoneal lavage cytology, which indicates unresectability, remains controversial. This study sought to determine whether positive peritoneal lavage cytology (CY+) precludes pancreatectomy. Furthermore, we propose a novel liquid biopsy using peritoneal lavage fluid to detect viable peritoneal tumor cells (v-PTCs) with TelomeScan F35, a telomerase-specific replication-selective adenovirus engineered to express green fluorescent protein. Resectable cytologically or histologically proven PDAC patients (n = 53) were enrolled. CY was conducted immediately following laparotomy. The resulting fluid was examined by conventional cytology (conv-CY; Papanicolaou staining and MOC-31 immunostaining) and by the novel technique (Telo-CY; using TelomeScan F35). Of them, 5 and 12 were conv-CY+ and Telo-CY+, respectively. All underwent pancreatectomy. The two double-CY+ (conv-CY+ and Telo-CY+) patients showed early peritoneal recurrence (P-rec) postoperatively, despite adjuvant chemotherapy. None of the three conv-CY+ Telo-CY− patients exhibited P-rec. Six of the 10 Telo-CY+ conv-CY− patients (60%) relapsed with P-rec. Of the remaining 38 double-CY− [conv-CY−, Telo-CY−, conv-CY± (Class III)] patients, 3 (8.3%) exhibited P-rec. Although conv-CY+ status predicted poor prognosis and a higher risk of P-rec, Telo-CY was more sensitive for detecting v-PTC. Staging laparoscopy and performing conv-CY and Telo-CY are needed to confirm the indication for pancreatectomy.

## Introduction

Despite the general trends towards increased cancer survival, pancreatic ductal adenocarcinoma (PDAC) has a dismal prognosis, with a 5-year survival rate of approximately 8%^1^. Indeed, PDAC is currently fourth on the list of leading causes of cancer-related mortality^1,2^; an estimated 64,050 people were diagnosed with PDAC in 2023, of whom approximately 50,550 were expected to die from this disease. By 2030, PDAC is predicted to become the second leading cause of cancer deaths in Western countries^1,3^. Though surgical resection of localized disease with negative margins (R0) provides the best opportunity for cure, only approximately 20% of PDAC patients have resectable or borderline-resectable disease, and most individuals develop tumor recurrence after pancreatectomy, with the median recurrence-free survival rate ranging from 6 to 23 months^4–6^. Recently, multidisciplinary treatment combined with neoadjuvant and adjuvant therapies, including chemo- and chemoradiation therapies, were developed and have been shown to improve survival^6–8^. However, up to 80% of PDAC patients who have undergone resection develop disease recurrence, despite the initial apparent curative efficacy of pancreatectomy and sequential adjuvant therapy^4,9^. In particular, recurrences that develop in the early postoperative period have been attributed to the presence of occult micrometastases beyond the margins of surgical resection at the time of surgery^10^.

Peritoneal recurrence is a major recurrence pattern, with an incidence ranging from 8 to 49%^6,11–14^. Accordingly, the resectability status of lesions, in combination with either preoperative or intraoperative diagnosis of tumor staging, is important for selecting the appropriate therapeutic strategy for each PDAC patient^15^. Peritoneal lavage cytology (CY) is an important diagnostic technique for assessing tumor progression. Positive peritoneal cytology is a strong predictor of the development of peritoneal recurrence^16^. According to the American Joint Committee on Cancer and the National Comprehensive Cancer Network, peritoneal fluid with microscopic evidence of tumor cells, known as CY-positive (CY+) status, is defined as M1 disease^15,17,18^. However, peritoneal recurrence occurs even in patients undergoing curative resection for localized PDAC and who exhibit negative cytology^14^. For instance, a noteworthy study reported that the frequency of peritoneal recurrence in CY-negative (CY−) patients was 21%^14^. This apparent inconsistency may be attributable to the lack of sensitivity of the conventional cytology examination, which uses Papanicolaou staining for the detection of minimal tumor cell dissemination in the peritoneal cavity. Collectively, these results demonstrate that the development of cytological methods with higher sensitivity is essential for predicting or detecting peritoneal micrometastasis.

In an effort to improve the diagnostic sensitivity of assays for the dissemination of minute tumor cell in the peritoneal cavity, several papers have reported novel methods using peritoneal lavage fluid. Takahashi et al., among others, reported that reverse transcription-polymerase chain reaction (RT-PCR)-based diagnostic tests are more sensitive than conventional cytological examination; these researchers found that RT-PCR+ status is significantly associated with peritoneal recurrence and impaired survival in patients with gastroenterological cancers (including gastric and colon cancers) and PDAC^19–21^. A recent study showed that the levels of carcinoembryonic antigen (CEA) and carbohydrate antigen 19-9 (CA19-9) in peritoneal lavage fluid are sensitive biomarkers for predicting or detecting peritoneal micrometastases^22–25^. Furthermore, a group at Tohoku University demonstrated that the testing of tumor-derived DNA, obtained in peritoneal lavage fluid from PDAC patients (i.e., “liquid biopsy”), is useful for identifying individuals likely to develop peritoneal dissemination; using this technique, these researchers assessed the minimal residual disease in peritoneal cavity associated with early peritoneal recurrence^26,27^.

Telomerase expression is a specific hallmark of cancer^28^ and is required for the unregulated proliferation of tumor cells. The catalytic domain of human telomerase, encoded by the *hTERT* gene, is silenced in normal somatic cells, but is activated in the majority of cancers^29^. In our previous study, we used TelomeScan F35, a telomerase-specific, replication-selective, green fluorescent protein (GFP)-expressing adenovirus system to specifically detect viable circulating tumor cells (v-CTCs) in blood samples obtained from patients with colorectal cancer^30,31^. Using serial detection by TelomeScan F35, we assessed the correlation between an increased v-CTC count and stent placement in patients with obstructive colorectal cancer (OCRC); our results clearly demonstrated a significant increase in the number of v-CTCs following stent insertion in patients with OCRC. In other words, treatment of OCRC by endoscopic stent insertion resulted in the dissemination of tumor cells into the peripheral blood circulation, which reduced the likelihood of survival. This work confirmed the utility of the TelomeScan F35 detection system for conducting a “liquid biopsy” using blood samples for visual detection of live human cancer cells.

In the present study, we assessed the clinical utility of high-quality testing for CY examination using the TelomeScan F35 detection system in PDAC patients. Specifically, we compared the findings obtained using this “Telo-CY” technique to those obtained with the conventional CY (conv-CY) examination.

## Results

### Patient demographics and tumor characteristics

Patient characteristics are summarized in Table 1. This study enrolled 53 patients between January 2015 and February 2019. The cohort included 30 males and 23 females, with a median age of 73 years (range, 53–87 years). The mean follow-up period after surgery was 35.4 months (range 7–102 months) or until death. Of the 53 patients, 40 (76%) underwent upfront surgery without any preoperative therapies. The other 13 (24%) patients underwent surgical resection after preoperative treatment consisting of gemcitabine plus S-1 (an oral fluoropyrimidine derivative) or gemcitabine plus S-1 plus radiation. Forty-four patients (83%) received adjuvant chemotherapy with S-1, whereas 9 patients (17%) lost the opportunity for postoperative adjuvant therapy due to their poor general condition (Table 1). Using conv-CY, malignant cells (conv-CY+) were detected in CY samples from five of the 53 patients; malignant cells were not detected in CY samples from another 46 patients (conv-CY−). CY samples from the remaining two patients were atypical category^32^ (i.e., Papanicolaou classification Class III), here termed conv-CY±. Using Telo-CY, viable malignant cells were detected in CY samples from 12 of 53 patients (Telo-CY+); samples from the other 41 patients were negative on the TelomeScan F35 assay (Telo-CY−). For the combined cytological diagnosis with both conv- and Telo-CY, 15 patients were classified as CY+, a group that consisted of samples that were positive on conv-CY and/or Telo-CY; the other 38 patients were categorized as CY−, a group that included individuals who were both conv-CY− and Telo-CY−, and those who were conv-CY± (Class III)^32^ (Tables 1 and 2).

**Table 1:** Patients’ clinicopathological characteristics.

Characteristics	No. of patients
Age, y (median, range)	73, 53–87
Sex (male/female)	30/23
Tumor diameter, mm (median, range)^a^	25, 6–65
Histopathological type (well/mod/poor)	18/27/8
Pathological depth of invasion pT (T1/T2/T3/T4) (UICC 8th Ed.)	3/8/42/0
Pathological lymph node metastasis pN (N0/N+) (UICC 8th Ed.)	21/32
Operation (PD/DP/TP)	32/14/7
CA19-9, U/L (mean** ± **SD, range)	888.6 ± 2187.1, 2–13,482
CEA, ng/mL (mean** ± **SD, range)	5.8 ± 6.8, 1.1–37.5
Preoperative treatment (NAC/NACRT/Upfront surgery)	3/10/40
Adjuvant chemotherapy (+/−)	44/9
Peritoneal lavage cytology (conventional-CY) (−/+/± = Class III)^b^	46/5/2
TelomeScan PTC by Telo-CY (−/+)	41/12
Combined cytological diagnosis (CY−/CY+) [CY− : conv-CY− and Telo-CY−, conv-CY±] [CY+: conv-CY+ and/or Telo-CY +]	38/15

*Well* well-differentiated type, *mod* moderately differentiated type; poor, poorly differentiated type, *CA19-9* carbonic anhydrase 19–9, *CEA* carcinoembryonic antigen, *PD* pancreaticoduodenectomy, *DP* distal pancreatectomy, *TP* total pancreatectomy, *pT* pathological primary tumor, *pN* pathological regional lymph nodes, *NAC* neoadjuvant chemotherapy, *NACRT* neoadjuvant chemoradiation therapy, *PTC* peritoneal tumor cell, *CY* peritoneal lavage cytology, *conv-CY* Papanicolaou-based conventional CY, *Telo-CY* TelomeScan-based CY, *UICC* Union for International Cancer Control, “TNM Classification of Malignant Tumors”, 8th edition.

^a^Tumor diameter was determined by computed tomography/magnetic resonance imaging.

^b^Papanicolaou classification (Papanicolaou Society of Cytopathology Guidelines 2014). CY−, Class II; CY+, Class VI and V; CY±, Class III (atypical).

**Table 2:** Comparison of clinicopathological characteristics of the patients in the CY− and CY+ groups.

Characteristics	CY− group (n = 38) (conv-CY− and Telo-CY−, conv-CV±)	CY+ group (n = 15) (conv-CY+ and/or Telo-CY+)	*P* value
Age, y (median, range)	70, 53–84	74, 57–87	0.097
Sex (male/female)	19/19	11/4	0.408
Tumor diameter, mm (< 20/ > 20) (median size)^a^	9/29 (25 mm)	5/10 (26 mm)	0.844
Histopathological type (well or mod/poor)	32/6	13/2	0.700
Pathological depth of invasion pT (T1 or T2/T3) (UICC 8th Ed.)	8/30	3/12	0.356
Pathological lymph node metastasis pN (N0/N1 or N2) (UICC 8th Ed.)	15/23	6/9	0.390
Pathological factors of primary tumors			
Invasion of the anterior pancreatic capsule (−/+)	17/21	5/10	0.697
Invasion of the retroperitoneal tissue (−/+)	11/27	4/11	0.304
Portal vein invasion (−/+)	22/16	12/3	0.110
Arterial invasion (−/+)	34/4	13/2	0.824
Bile duct invasion (−/+)	24/14	10/5	0.675
Duodenal invasion (−/+)	27/11	10/5	0.808
Perineural invasion (−/+)	8/30	3/12	0.810
Lymph vessel invasion (−/+)	21/17	7/8	0.778
Vascular invasion (−/+)	11/27	5/10	0.433
Operation (PD/DP/TP)	22/11/5	10/3/2	0.533
CA19-9, U/L (mean ± SD, range)	927.7 ± 2391.5 (2–13,482)	688.9 ± 1240.4 (5–4761)	0.845
CEA, ng/mL (mean ± SD, range)	6.3 ± 7.7 (1.8–38)	4.3 ± 2.5 (1.1–10.4)	0.083
Preoperative treatment (NAC or NACRT) +/−)	10/28	3/12	0.381
Adjuvant chemotherapy (+/−)	32/6	12/3	0.597

*Well* well-differentiated type; mod, moderately differentiated type, *poor* poorly differentiated type; CA19-9, carbonic anhydrase 19-9, *CEA* carcinoembryonic antigen, *PD* pancreaticoduodenectomy, *DP* distal pancreatectomy, *TP* total pancreatectomy, *pT* pathological primary tumor, *pN* pathological regional lymph nodes, *NAC* neoadjuvant chemotherapy, *NACRT* neoadjuvant chemoradiation therapy, *CY* peritoneal lavage cytology, *conv-CY* Papanicolaou-based conventional CY, *Telo-CY* TelomeScan-based CY, The CY− group included patients who were conv-CY− and Telo-CY−, as well as the conv-CY± (Class III) patients. The CY+ group included patients who were conv-CY+ and/or Telo-CY+. Pathological examinations were performed in accordance with the Union for International Cancer Control (UICC) TNM Classification of Malignant Tumors, 8th edition. The clinicopathological parameters were compared using Fisher’s exact test.

^a^Tumor diameter was determined by computed tomography/magnetic resonance imaging.

### Comparison of clinicopathological factors between the CY+ and CY− groups

No significant differences in demographic characteristics were observed between the CY+ and CY− groups, specifically in regard to the following parameters: age; sex; any of the assessed tumor attributes, including tumor diameter, histopathological type, depth of invasion, lymph node metastasis, invasion of the anterior pancreatic capsule or retroperitoneal tissue, portal vein invasion, arterial invasion, perineural invasion, lymph vessel invasion, or vascular invasion; serum levels of CEA or CA19-9; or pre-/postoperative treatment (Table 2).

### Prognostic effects of peritoneal lavage cytology, including conventional and TelomeScan cytology

Table 3 shows the detailed results of both conv-CY and Telo-CY, along with prognoses. Two patients tested double-CY+, and another 3 patients tested positive only on conv-CY. In contrast, 10 patients were diagnosed positive only on Telo-CY. Two patients who tested conv-CY± (Class III) also tested Telo-CY−. The remaining 36 patients were double-CY−.

**Table 3:** Detailed results of peritoneal lavage cytology with conv-CY and Telo-CY, expression of tumor markers on v-PTCs, and presence or absence of peritoneal recurrence.

Combined cytological diagnosis (n)	Conv-CY diagnosis: +/− (MOC-31 staining: +/−)	Telo-CY diagnosis: +/− (Cell number of v-PTCs)	Expression of tumor markers on v-PTCs (Ep-CAM/CEA/CA19-9)	P-rec^a^ (Yes/No) (time for P-rec after operation) [survival time (Mo)]
Double-CY+ (2)	Conv-CY: + (MOC-31:+)	Telo-CY: + (16)	Ep-CAM+/CEA−/CA19-9+	Yes (7 Mo) [7 Mo]
	Conv-CY: + (MOC-31:+)	Telo-CY: + (6)	Ep-CAM+/CEA−/CA19-9+	Yes (9 Mo) [9 Mo]
Conv-CY+ only (3)	Conv-CY: + (MOC-31:+)	Telo-CY: − (0)	Ep-CAM−/CEA−/CA19-9−	No [14 Mo]
	Conv-CY: + (MOC-31: −) Large N/C ratio	Telo-CY: − (0)	Ep-CAM−/CEA−/CA19-9−	No [50 Mo]
	Conv-CY: + (MOC-31:+)	Telo-CY: − (0)	Ep-CAM−/CEA−/CA19-9−	No [20 Mo]
Telo-CY+ only (10)	Conv-CY: − (MOC-31: −)	Telo-CY: + (100)	Ep-CAM+/CEA−/CA19-9−	No [101 Mo]
	Conv-CY: − (MOC-31: −)	Telo-CY: + (44)	Ep-CAM−/CEA+/CA19-9−	No [88 Mo]
	Conv-CY: − (MOC-31: −)	Telo-CY: + (2)	Ep-CAM+/CEA−/CA19-9: N.D	Yes (14 Mo) [14 Mo]
	Conv-CY: − (MOC-31: −)	Telo-CY: + (2)	Ep-CAM−/CEA+/CA19-9: N.D	No [42 Mo]
	Conv-CY: − (MOC-31: −)	Telo-CY: + (9)	Ep-CAM+/CEA−/CA19-9+	Yes (42 Mo) [52 Mo]
	Conv-CY: − (MOC-31: −)	Telo-CY: + (6)	Ep-CAM+/CEA−/CA19-9: N.D	Yes (14 Mo) [14 Mo]
	Conv-CY: − (MOC-31: −)	Telo-CY: + (28)	Ep-CAM−/CEA+/CA19-9−	No [62 Mo]
	Conv-CY: − (MOC-31: −)	Telo-CY: + (39)	Ep-CAM+/CEA−/CA19-9−	Yes (18 Mo) [40 Mo]
	Conv-CY: − (MOC-31: −)	Telo-CY: + (19)	Ep-CAM+/CEA−/CA19-9+	Yes (8 Mo) [8 Mo]
	Conv-CY: − (MOC-31: −)	Telo-CY: + (7)	Ep-CAM+/CEA−/CA19-9−	Yes (8 Mo) [8 Mo]
Conv-CY ± (class III) (2)	Conv-CY: ± (MOC-31: −) Large N/C ratio	Telo-CY: − (0)	Ep-CAM−/CEA−/CA19-9−	No [46 Mo]
	Conv-CY: ± (MOC-31: −) Large N/C ratio	Telo-CY: − (0)	Ep-CAM−/CEA−/CA19-9−	No [18 Mo]
Double-CY− (36)	Conv-CY: − (MOC-31: −)	Telo-CY: − (0)	Ep-CAM−/CEA−/CA19-9−	Yes (3 cases) (7, 7, 24 Mo) [36 Mo]

Double-CY+ were patients positive on both conv-CY and Telo-CY (n = 2). Conv-CY+ only were patients diagnosed positive only on conv-CY (n = 3). Telo-CY+ only were patients diagnosed positive only on Telo-CY (n = 10). Conv-CY± were patients diagnosed as having atypical cells (Class III) on conv-CY but not Telo-CY− (n = 2). Double-CY− were patients diagnosed negative on both conv-CY and Telo-CY (n = 36).

*v-PTCs* viable peritoneal tumor cells, *CY* peritoneal lavage cytology, *conv-CY* Papanicolaou-based conventional CY, *Telo-CY* TelomeScan-based CY, *CA19-9* carbonic anhydrase 19-9, *CEA* carcinoembryonic antigen, *Ep-CAM* epithelial cell adhesion molecule (CD326), *N/C ratio* nucleus/cytoplasm ratio; *MOC-31 staining* immunostaining by anti-human epithelial-related antigen (clone MOC-31) monoclonal Ab, *CD45* (i.e. leukocyte common antigen, protein tyrosine phosphatase type C: PTPRC), *GFP* green fluorescence protein; *N.D.* not done.

^a^P-rec, peritoneal recurrence.

Regarding the possible association between combined cytology results and prognosis, we observed that the 2 patients who were double-CY+ relapsed with peritoneal recurrences at 7–9 months after surgical resection, despite curative surgery. In contrast, the 3 patients who were positive only on conv-CY exhibited significantly longer survival (P = 0.017) without peritoneal recurrence compared to patients who were double-CY+, with mean survival times [MSTs] of 28.7 months (conv-CY+ only) and 6.6 months (double-CY+). However, 6 of 10 patients who were positive only on Telo-CY died following peritoneal recurrences, with mortalities occurring 8–42 months after curative surgery (MST, 23.5 months) (Table 3). Collectively, these results indicated that the sensitivity for prediction of peritoneal recurrence was significantly higher with Telo-CY than conv-CY (P = 0.031). For double-CY− patients, only 3 of 36 patients exhibited recurrence with peritoneal dissemination, which was seen 7–24 months after surgical resection. The 2 patients who tested conv-CY± (Class III) and Telo-CY− survived without peritoneal recurrence (MST, 32.0 months). One of the two conv-CY± Telo-CY− patients remained alive at 46 months postoperatively; the other conv-CY± Telo-CY− patient exhibited relapse with liver metastasis 10 months postoperatively and died 18 months postoperatively (Table 3).

Supplemental Table 1 shows the detailed results of conv-CY and Telo-CY. For Telo-CY, the actual number of GFP+/CD45− cells and cell viability of those recovered in peritoneal lavage fluid are displayed.

Supplemental Fig. 1 summarizes the peritoneal recurrence rate in each cytological diagnostic group.

**Figure 1:**
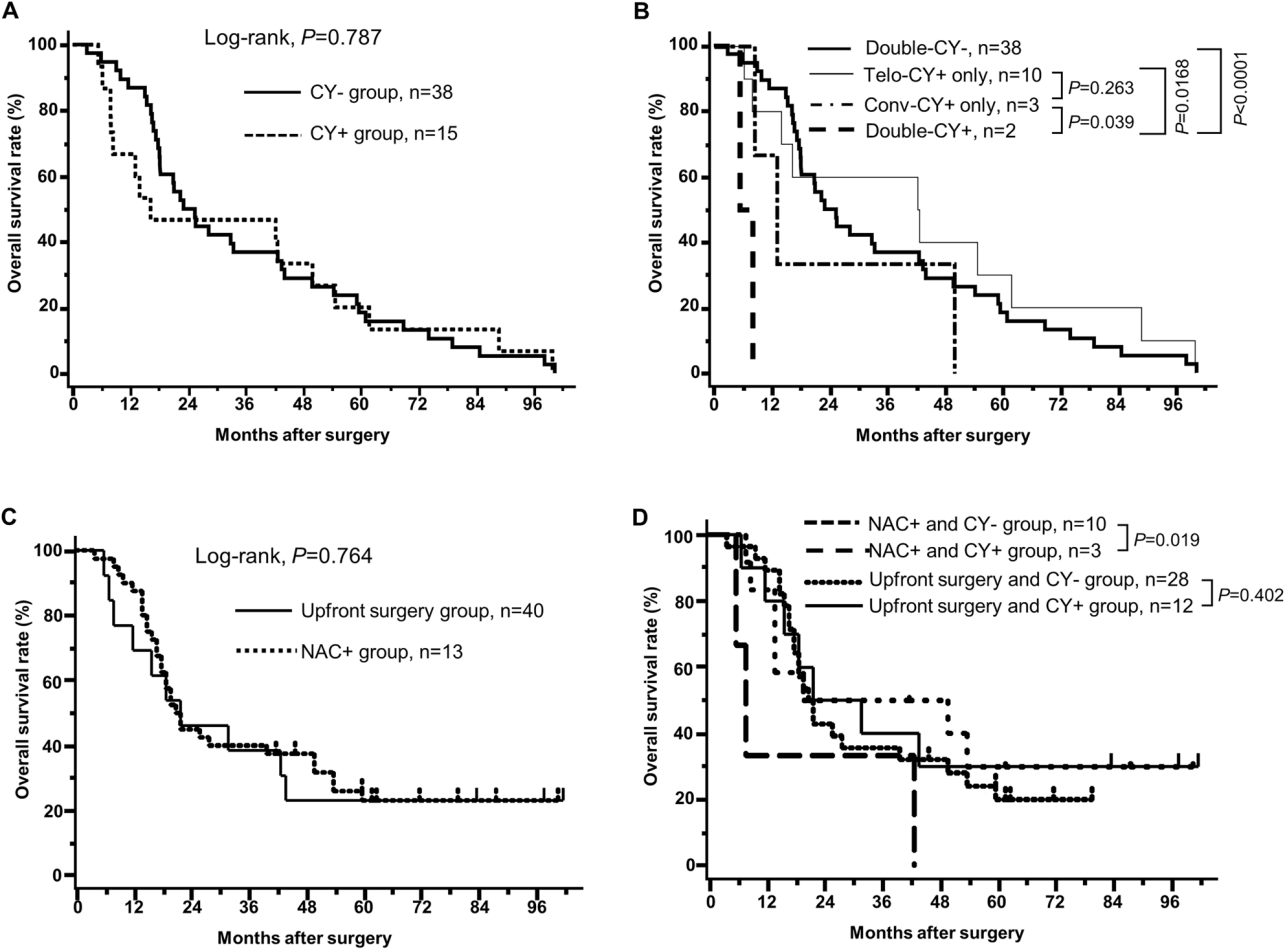
Kaplan–Meier plots of overall survival curves according to the conv-CY and Telo-CY results and subgroup analysis of the upfront surgery and neoadjuvant chemotherapy groups. (**A**) Kaplan–Meier plots of overall survival curves of the patients categorized into CY− and CY+ groups. The CY− group includes patients who were conv-CY− and Telo-CY−, as well as the conv-CY± (Class III) patients (n = 38). The CY+ group includes patients who were conv-CY+ and/or Telo-CY+ (n = 15) (P-value, log-rank test). (**B**) Kaplan–Meier plots of overall survival curves of the patients classified into four groups according to the conv-CY and Telo-CY results. The double-CY− group includes patients who were conv-CY− and Telo-CY−, as well as conv-CY± patients (n = 38). The Telo-CY+ alone group includes patients diagnosed positive only on Telo-CY (n = 10). The Conv-CY+ alone group includes patients diagnosed positive only on conv-CY (n = 3). The double-CY+ group includes patients positive on both conv-CY and Telo-CY (n = 2) (P-value, log-rank test). (**C**) Kaplan–Meier plots of overall survival curves of the patients treated with upfront surgery or neoadjuvant chemotherapy. The upfront surgery group includes patients who underwent pancreatectomy without neoadjuvant chemotherapy (n = 40). The NAC+ group includes patients who underwent pancreatectomy after NAC (n = 13) (P-value, log-rank test). (**D**) Kaplan–Meier plots of overall survival curves of the patients classified into four subgroups according to CY results, upfront surgery, and with or without NAC. Ten patients are in the NAC and CY− group, 3 patients are in the NAC and CY+ group, 28 patients are in the upfront surgery and CY− group, and 12 patients are in the upfront surgery and CY+ group (P-value, log-rank test).

### Association with peritoneal recurrence and appearance of tumor markers on v-PTCs

To distinguish cancer cells, cells were stained for tumor markers, including the epithelial cell adhesion molecule (Ep-CAM, aka CD326), CEA, and CA19-9, as described in “Materials and Methods”. For the 2 patients who were double-CY+, v-PTCs in the peritoneal lavage fluid clearly expressed both Ep-CAM and CA19-9; this is consistent with the peritoneal dissemination observed shortly after surgery (Table 3). Of the 10 patients who were positive only on Telo-CY, Ep-CAM-displaying v-PTCs were obtained from 6 patients, all of whom subsequently relapsed with peritoneal recurrence. In contrast, Ep-CAM was not expressed by the v-PTCs obtained from 3 of the other 4 patients; none of these 4 individuals developed peritoneal recurrence. These results indicated that Ep-CAM expression by v-PTCs contributes to the oncological potential of such cells; this observation is consistent with the known roles of this marker, including cell–cell adhesion and tumor cell proliferation, processes expected to lead to peritoneal dissemination^33^.

### Association of peritoneal lavage cytology status with overall survival

Figure 1a shows the overall survival (OS) curves of the PDAC patients in the CY+ and CY− groups. The OS of patients who tested CY+ or CY− did not differ significantly [MSTs of 34.5 months (CY+) and 35.8 months (CY−); *P* = 0.785]. In contrast, the OS of the 2 patients who tested double-CY+ was significantly shorter than that of the 38 patients who tested double-CY− (MST, 6.6 months (double-CY+) and 35.8 months (double-CY−); *P* < 0.0001; Fig. 1b). Similarly, the OS of the double-CY+ patients (MST, 6.6 months) was significantly shorter than of patients who tested either positive only on conv-CY (MST, 28.7 months; *P* = 0.039) or positive only on Telo-CY (MST, 43.4 months; *P* = 0.017) (Fig. 1b). No significant differences were observed in the comparison of OS between patients who tested positive only on conv-CY and positive only on Telo-CY (MST, 28.7 vs. 43.4 months, respectively; *P* = 0.263) (Fig. 1b).

As for the prognostic effects of neoadjuvant chemotherapy (NAC) on the CY+ and CY− groups, the OS of the 13 patients who received NAC was similar to that of the 40 patients who did not receive NAC (upfront surgery group) [MST, 37.9 months (NAC+ group) and 34.5 months (upfront surgery group); P = 0.764] (Fig. 1c). In contrast, in the subgroup analysis between the NAC+ and CY− and the NAC+ and CY+ groups, significant improvement in OS was observed in the NAC+ and CY− subgroup [MST, 43.6 months (NAC+ and CY−) and 19.0 months (NAC+ and CY+); P = 0.019] (Fig. 1d). Furthermore, whereas all 3 patients who received NAC with still CY+ status relapsed with peritoneal recurrence, only one of the 10 patients treated with NAC with CY− status relapsed with peritoneal recurrence. These results suggested that NAC could prevent relapse with peritoneal recurrence and improve OS. In the subgroup analysis between the NAC− and CY+ and the NAC− and CY− groups, no significant difference was found [MST, 39.7 months (NAC− and CY+) and 32.4 months (NAC− and CY−); P = 0.402] (Fig. 1d).

In addition, the prognostic effects of adjuvant chemotherapy on the CY+ and CY− groups were further analyzed. The OS of the 44 patients who received adjuvant chemotherapy was longer than that of the 9 patients who did not receive adjuvant chemotherapy [40.0 months (adjuvant+) and 27.6 months (adjuvant-); *P* = 0.279]. Unfortunately, no significant difference was observed between the adjuvant chemotherapy+ and adjuvant chemotherapy− groups. In the subgroup analysis between the CY+ and adjuvant chemotherapy+ and the CY− and adjuvant chemotherapy+ groups, no significant improvement in OS was observed [MST, 38.0 months (CY+ and adjuvant chemotherapy+) and 36.5 months (CY− and adjuvant chemotherapy+); P = 0.895]. In the subgroup analysis between the CY+ and adjuvant chemotherapy− and the CY− and adjuvant chemotherapy− groups, no significant difference in OS was found [MST, 24.0 months (CY+ and adjuvant chemotherapy−) and 29.3 months (CY− and adjuvant chemotherapy−); P = 0.875].

### Representative findings of peritoneal lavage cytology, analyzed by either conventional-CY or TelomeScan-CY

To clarify the results of peritoneal lavage cytology analyzed using the two methods, we provide representative findings for three classes of patients.

**Figure 2:**
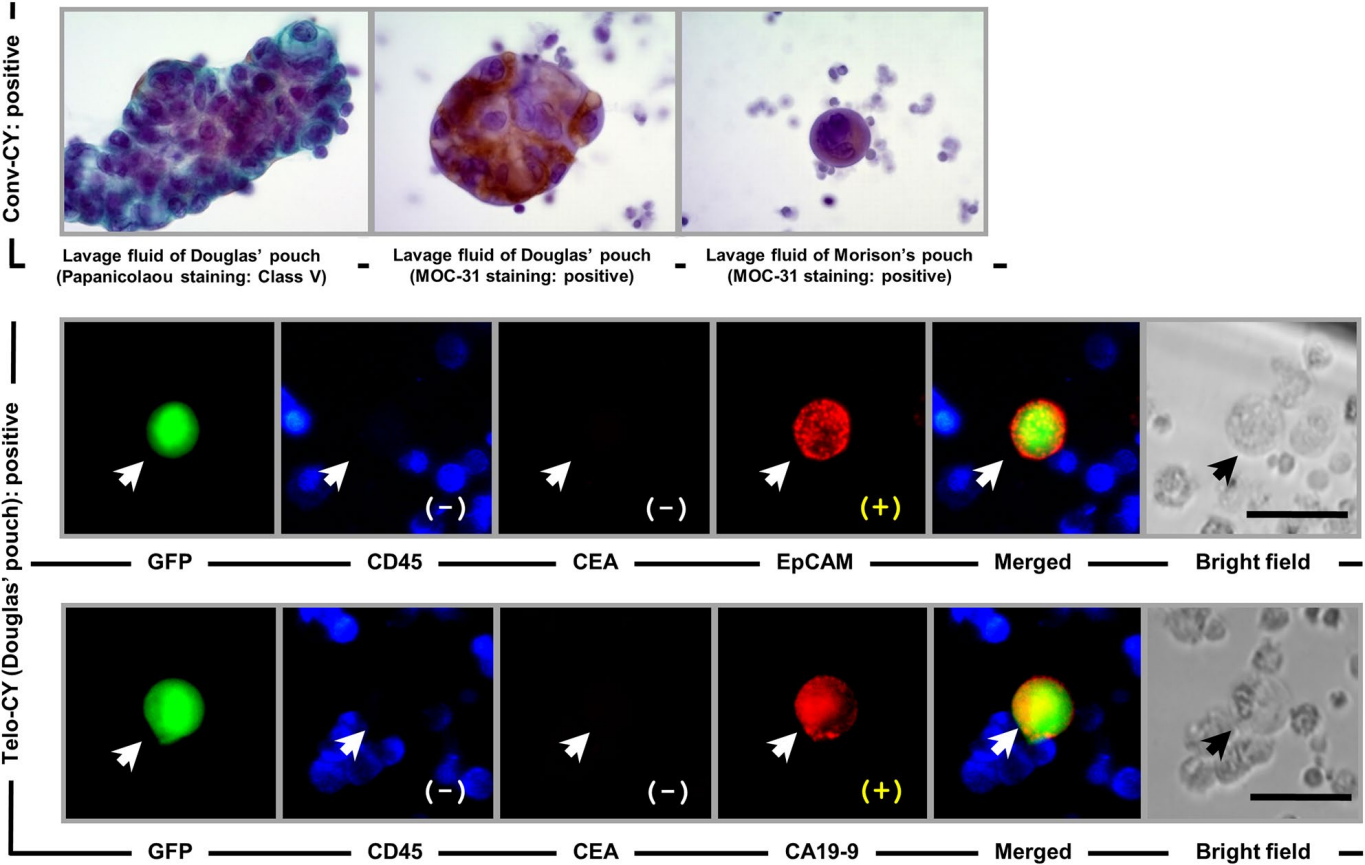
Representative CY findings of a double-CY+ patient. Top: conv-CY findings, stained with Papanicolaou and immunostained with MOC-31. Bottom: Telo-CY findings, including GFP fluorescence; immunocytochemical staining of CD45, CEA, CA19-9, or Ep-CAM; merged fluorescence; and bright-field image. CY, peritoneal lavage cytology; conv-CY, Papanicolaou-based conventional CY; Telo-CY, TelomeScan-based CY; CA19-9, carbonic anhydrase 19-9; CEA, carcinoembryonic antigen; Ep-CAM, epithelial cell adhesion molecule (CD326); MOC-31 staining, immunostaining by anti-human epithelial-related antigen (clone MOC-31) monoclonal Ab; CD45 (i.e., leukocyte common antigen, protein tyrosine phosphatase type C: PTPRC); GFP, green fluorescence protein. White and black arrows show v-PTCs. Scale bar: 30 μm.

Figure 2 shows the cytological and immunocytochemical findings from double-CY+ patients. In lavage fluid recovered from the pouch of Douglas, conv-CY detected adenocarcinoma cells arranged in a syncytial fashion, for which the cell boundaries were not clearly resolved. On Papanicolaou staining, these findings were defined as Class V. Furthermore, conv-CY detected small aggregates of cancer cells arranged in a ball-like pattern; these clusters stained positive for MOC-31. On Telo-CY, the v-PTCs detected by TelomeScan F35 tested GFP+ and CD45− and demonstrated expression of CA19-9 and Ep-CAM, but not of CEA (Fig. 2, white arrow). For bright-field findings, the morphology of these v-PTCs was similar to that of other cells, such as reactive mesothelial cells, also present in the peritoneal lavage fluid. As noted above, patients who tested double-CY+ relapsed with peritoneal recurrence 7 months after surgical resection.

**Figure 3:**
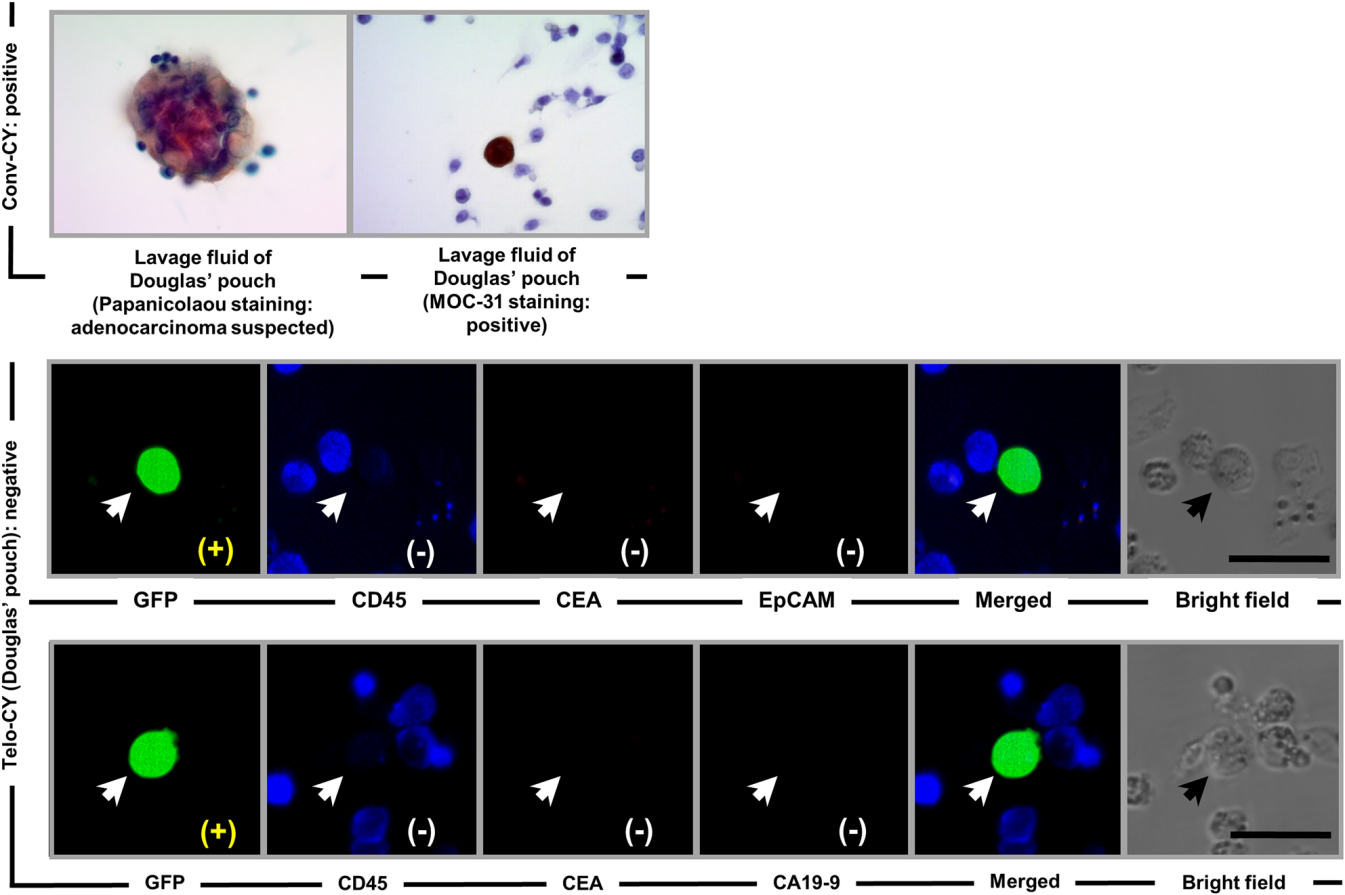
Representative CY findings of a patient positive on conv-CY alone. Top: conv-CY findings, stained with Papanicolaou and immunostained with MOC-31. Bottom: Telo-CY findings, including GFP fluorescence; immunocytochemical staining of CD45, CEA, CA19-9, or Ep-CAM; merged fluorescence; and bright-field image. CY, peritoneal lavage cytology; conv-CY, Papanicolaou-based conventional CY; Telo-CY, TelomeScan-based CY; CA19-9, carbonic anhydrase 19-9; CEA, carcinoembryonic antigen; Ep-CAM, epithelial cell adhesion molecule (CD326); MOC-31 staining, immunostaining by anti-human epithelial-related antigen (clone MOC-31) monoclonal Ab; CD45 (i.e. leukocyte common antigen, protein tyrosine phosphatase type C: PTPRC); GFP, green fluorescence protein. White and black arrows show v-PTCs. Scale bar: 30 μm.

Figure 3 shows the cytological findings for a patient who was positive only on conv-CY. Conv-CY detected small clusters of cells that were arranged in a ball-like pattern and stained positively for MOC-31 staining; these findings were similar to those observed in lavage-derived cancer cells detected in patients who were double-CY+, as described above (Fig. 2). However, on Telo-CY, the viable cells were GFP+ and CD45− (Fig. 3, white arrow), whereas none of the tested tumor markers (including CEA, CA19-9, and Ep-CAM) were expressed on GFP+ cells (Fig. 3, white arrow). Therefore, the viable lavage-derived cells could not be regarded as viable cancer cells on Telo-CY. This patient, who tested positive on conv-CY alone, did not relapse with postoperative peritoneal recurrence of PDAC (Fig. 3).

**Figure 4:**
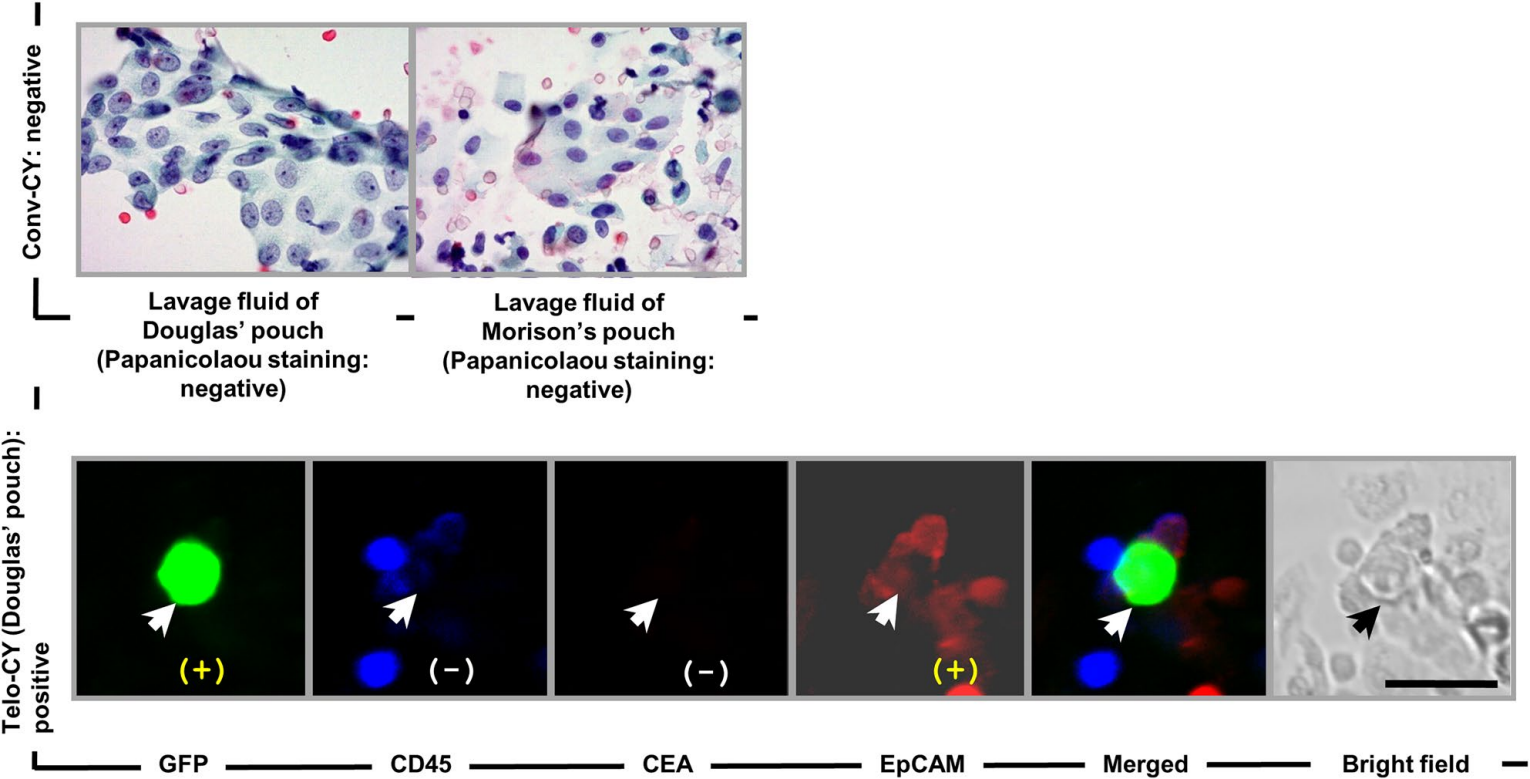
Representative CY findings of a patient positive on Telo-CY alone. Top: conv-CY findings, stained with Papanicolaou staining. Bottom: Telo-CY findings, including GFP fluorescence; immunocytochemical staining of CD45, CEA, or Ep-CAM; merged fluorescence; and bright-field image. CY, peritoneal lavage cytology; conv-CY, Papanicolaou-based conventional CY; Telo-CY, TelomeScan-based CY; CEA, carcinoembryonic antigen; Ep-CAM, epithelial cell adhesion molecule (CD326); CD45 (i.e. leukocyte common antigen, protein tyrosine phosphatase type C: PTPRC); GFP, green fluorescence protein. White and black arrows show v-PTCs. Scale bar: 30 μm.

Figure 4 shows the cytological findings for a patient who tested positive only on Telo-CY. On Papanicolaou staining in conv-CY, cells from this individual did not display any malignant findings [e.g., no large nucleus/cytoplasm (N/C) ratio and no nuclear atypia]. Accordingly, these cells were defined as Class II based on the Papanicolaou classification system^32^. In contrast, Telo-CY of viable cells from this patient was GFP+ and CD45− and demonstrated expression of Ep-CAM, but not of CEA (Fig. 4, white arrow). Therefore, the viable cells recovered in peritoneal lavage fluid and detected by TelomeScan F35 were judged to be viable cancer cells. Consistent with these findings, this patient exhibited peritoneal dissemination 12 months postoperatively, and then died 14 months postoperatively.

Further representative CY findings of a patient class III on conv-CY and Telo-CY negative are shown in supplemental Fig. 2.

## Discussion

Even “localized” PDAC, as diagnosed by radiographic examination, displays a tendency to result in advanced systemic disease; indeed, the majority of patients with localized disease already possess occult metastatic disease, including distant and peritoneal tumor spread, even though such metastases are radiographically undetectable at the time of the initial diagnosis^34^. Therefore, pancreatectomy is not oncologically beneficial for patients with apparently localized PDAC. To better discriminate patients who are likely to benefit from surgery from those who are not, novel imaging- or molecular-based assessments will need to be developed; such techniques must have increased sensitivity for the detection of minute metastases, thereby facilitating the precise staging of tumors. To address the high incidence of cancer recurrence in PDAC patients, who typically are treated by upfront surgery alone, several multidisciplinary treatment strategies have been implemented^35–38^. Preoperative chemo- and chemoradiation therapies are promising modalities for the treatment of PDAC^34,36–38^; in patients with resectable and borderline-resectable PDAC who were subsequently treated with these preoperative therapies, the 5-year survival rates were markedly increased^35–38^. Nonetheless, a substantial number of patients developed peritoneal recurrence despite having undergone perioperative treatment^37^.

Peritoneal recurrence is a major recurrence pattern, and the survival of patients with peritoneal recurrence has been reported to be worse than that of patients with other sites of recurrence^14^. Since cancer cells exfoliated from primary PDAC tumors are thought to be the precursors of peritoneal implantation^39–41^, preoperative and intraoperative detection of malignant cells in the peritoneal cavity is of great importance for the prediction of early peritoneal recurrence after surgery.

Peritoneal lavage cytology (CY) conventionally has been conducted using the Papanicolaou and Giemsa staining techniques^42^. Papanicolaou staining is globally accepted and used as a standard method in cytology. Although cytological evaluation of peritoneal lavage fluid obtained from PDAC patients is conducted primarily by these methods^16,43–45^, CY by Papanicolaou staining may lead to false-negative results in some cancer patients^46^. These false-negative results may reflect the small number of cancer cells recovered in the peritoneal lavage fluid. Alternatively, the heteromorphism of epithelial cells is weak and difficult to diagnose as malignant in some PDAC cases. Accordingly, additional immunocytochemical staining, such as that for CEA, may facilitate cancer detection when the number of cancer cells is small or it is the staining that is weak or atypical, rather than the cells themselves^42,46–48^. However, in the present study, in which the sensitivity of Papanicolaou-based conv-CY was augmented by the inclusion of immunohistochemical staining for MOC-31, conv-CY provided detection in only 40% of patients who subsequently exhibited peritoneal recurrence (Table 3; Supplemental Fig. 1). The identification of malignant cells using Papanicolaou-based conv-CY augmented by immunocytological staining still may have been limited by the small number of cancer cells available for slide preparation and staining. Furthermore, these cytological results, as obtained by a combination of conventional and immunohistochemical CY methods, do not reflect the viability of cancer cells; such cells must remain viable to participate in peritoneal dissemination following pancreatectomy. In other words, the cancer cells recovered in peritoneal lavage fluid will be diagnosed as “CY+” on conv-CY, whereas malignant cells exfoliated from primary PDAC tumors are destined to die soon due to the treatments with preoperative chemo- or chemoradiation therapies. Collectively, these observations suggest the prediction of peritoneal recurrence after surgery necessitates the development of high-quality testing for CY; such tests will need to specifically detect malignant cells, as well as their viability.

CTCs are promising biomarkers in several cancers^49–51^. However, CTCs are rare in peripheral blood, with numbers as low as one CTC per 10^6^–10^7^ leukocytes. Although several methods have been developed for the detection of CTCs^52,53^, such conventional CTC detection techniques present several problems. For instance, in the Cell Search™ system, which was approved by the US Food and Drug Administration in 2004, CTCs are concentrated using anti-CD45 and anti-Ep-CAM antibodies, and they are subsequently detected by immune staining using antibodies against cytokeratin (CK)-8, CK-9, and CK-19^54^. However, these antigens are also expressed on normal epithelial cells^55^. As a result, the cells detected as CTCs include a substantial number of false positives^56^. In response to these concerns, a novel viable CTC detection system was recently developed^57^ using a GFP-encoding conditionally replicating adenovirus (rAd-GFP). Furthermore, Kojima and Fijiwara reported previously that intratumoral injection of telomerase-specific replication-selective adenovirus expressing the GFP gene (OBP-401; TelomeScan) causes viral spread into the regional lymphatics, with subsequent specific replication in neoplastic lesions, resulting in GFP expression in metastatic lymph nodes in nu/mu mice. Collectively, rAd-GFP (OBP-401; TelomeScan) can replicate and express GFP fluorescence only in viable cancer cells. Subsequently, to further decrease the proportion of false-positive results, Sakurai et al. developed a novel, conditionally replicating adenovirus (rAdF35-142 T-GFP, aka TelomeScan F35) for specific detection of CTCs without false positives^30,31^. Collectively, rAd-GFP possesses a human telomerase reverse transcriptase (*hTERT*) promoter-driven E1 gene expression cassette and GFP expression cassette in the E1- and E3-deleted regions of the Ad genome, respectively. Incubation of rAd-GFP with blood cells containing cancer cells (i.e., CTCs) results in efficient labeling of cancer cells with GFP, because expression levels of *hTERT* are upregulated in most cancer cells^28^. However, production of false-positive cells (GFP-positive normal blood cells) was found when using rAd-GFP, particularly at high titers. Moreover, there is another drawback to the CTC detection method using rAd-GFP. CTCs lacking or expressing low levels of Coxsackievirus-adenovirus receptor (CAR) cannot be detected by this adenovirus. To suppress the production of false-positive cells, sequences perfectly complementary to blood cell-specific microRNA, miR-142-3p, were incorporated into the 3’-untranslated regions of the E1B and GFP genes. In addition, the fiber protein was replaced with that of Ad serotype 35, which recognizes human CD46, creating rAdF35-142 T-GFP (OBP-1101; TelomeScan F35). TelomeScan F35 could overcome these obstacles to specific detection of cancer cells by the improvements described above. In the present study, we used TelomeScan F35 to detect v-PTCs in peritoneal lavage fluid obtained from PDAC patients.

**Figure 5:**
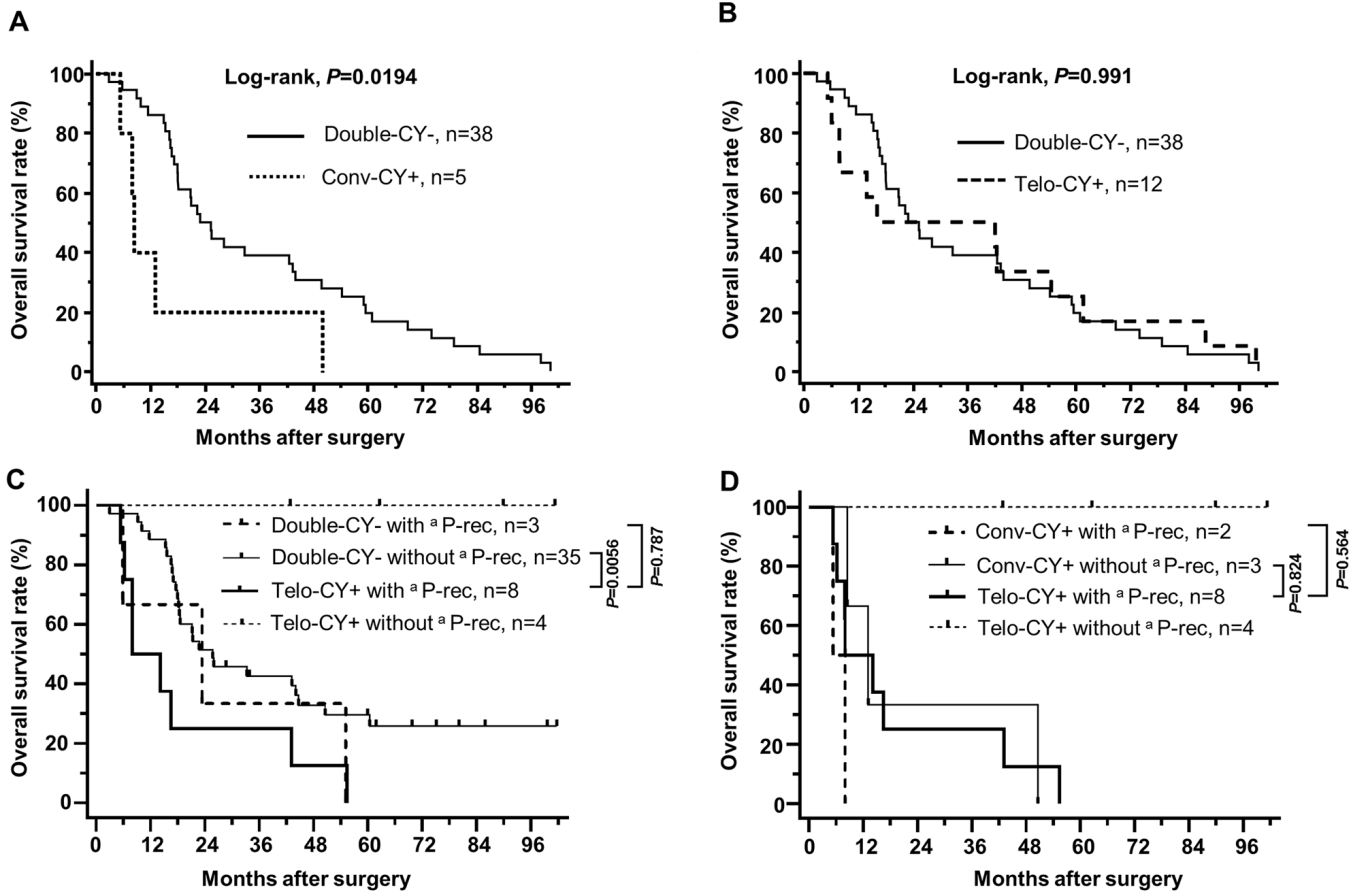
Kaplan–Meier plots of overall survival curves for subgroup analysis. (**A**) Kaplan–Meier plots of overall survival curves of the patients, categorized based on double-CY− vs. conv-CY+ status (P-value, log-rank test). Double-CY-negative patients are conv-CY− and Telo-CY−, as well as conv-CY± patients (n = 38). Conv-CY-positive patients are positive only on conv-CY (n = 3) and double-CY-positive (n = 2). (**B**) Kaplan–Meier plots of overall survival curves of the patients, categorized based on double-CY− vs. Telo-CY+ status (P-value, log-rank test). Double-CY-negative patients are as described above (n = 38). Telo-CY-positive patients are positive only on Telo-CY (n = 10) and double-CY-positive (n = 2). (**C**) Kaplan–Meier plots of overall survival curves of the patients, categorized based on double-CY− vs. Telo-CY+ status and exhibiting relapse with or without peritoneal dissemination (P-value, log-rank test). Double-CY-negative patients are as described above (n = 38). Double-CY-negative with peritoneal recurrence (n = 3), double-CY-negative without peritoneal recurrence (n = 35), Telo-CY-positive with peritoneal recurrence are positive only on Telo-CY with peritoneal recurrence after surgery (n = 6), and double-CY-positive (n = 2). Telo-CY-positive without peritoneal recurrence (n = 4). (**D**) Kaplan–Meier plots of overall survival curves of the patients, analyzed based on conv-CY+ vs. Telo-CY +sstatus and relapsed with or without peritoneal dissemination (P-value, log-rank test). Conv-CY-positive patients are as described above (n = 5). Conv-CY-positive with peritoneal recurrence (n = 2), Conv-CY-positive without peritoneal recurrence (n = 3), Telo-CY-positive with peritoneal recurrence patients are as described above (n = 8). Telo-CY-positive without peritoneal recurrence (n = 4). ^a^P-rec, peritoneal recurrence.

Regarding the association between CY status and survival outcome (Figs. 1 and 5), we found that CY+ status, whether conv-CY+ and/or Telo-CY+, did not affect patient survival (Fig. 1a). Nonetheless, the OS of 5 patients who tested conv-CY+, including 2 double-CY+ individuals and 3 who were positive only on conv-CY, was significantly shorter than that of 38 patients who were double-CY− [MST, 16.4 months (conv-CY+) and 35.8 months (double-CY−); P = 0.0194)] (Fig. 5a). In contrast, the survival outcome in 12 patients with Telo-CY+ status, including 2 double-CY+ individuals and 10 positive on Telo-CY alone, did not differ significantly from that of double-CY− patients [MST, 37.2 months (Telo-CY+) and 35.8 months (double-CY−); P = 0.991) (Fig. 5b)]. Nonetheless, 8 of the 12 patients who tested positive on Telo-CY relapsed with peritoneal recurrence (Table 3; Supplemental Fig. 1), along with an OS that was significantly shorter than that of the 35 patients who were double-CY− and relapsed without peritoneal recurrence [MST, 19.3 months (Telo-CY+ with peritoneal recurrence) and 36.0 months (double-CY− without peritoneal recurrence); P = 0.0056] (Fig. 5c). In contrast, no significant differences were observed in survival outcomes between the 8 patients who tested positive on Telo-CY with peritoneal recurrence and the 3 patients who were double-CY− with peritoneal recurrence [MST, 19.3 months (Telo-CY+ with peritoneal recurrence) and 27.7 months (double-CY− with peritoneal recurrence); P = 0.787] (Fig. 5c). Four patients who tested positive on Telo-CY without peritoneal recurrence survived for a remarkably long term. These four patients were supposedly false-positive patients on Telo-CY assessment, but this adverse result may be overcome by Telo-CY assessment combined with testing for Ep-CAM expression, as described in detail below. In further subgroup analysis in regard to cytological diagnostic group with or without peritoneal recurrence, the survival outcome of 8 patients who tested positive on Telo-CY and relapsed with peritoneal recurrence did not differ significantly from that of the patients who were conv-CY+ with peritoneal recurrence (double-CY+ patients) [MST, 19.3 months (Telo-CY+ with peritoneal recurrence) and 6.6 months (conv-CY+ with peritoneal recurrence; double-CY+ patients); P = 0.564] (Fig. 5d). In addition, the survival outcome of 8 patients who tested positive on Telo-CY and relapsed with peritoneal recurrence also did not differ significantly from that of the patients who were conv-CY+ without peritoneal recurrence [MST, 19.3 months (Telo-CY+ with peritoneal recurrence) and 28.0 months (conv-CY+ without peritoneal recurrence); P = 0.824]. These results suggested that Telo-CY detected cancer cells capable of dissemination into the abdominal cavity and adversely affecting survival outcomes, even in patients for whom the results of conv-CY were negative. Notably, though positive results on conv-CY indicate the likely spread of cancer cells from PDAC tumors into the peritoneal cavity, conv-CY alone may not be sufficient to identify all such patients, especially for samples in which the cancer cells recovered by peritoneal lavage are small in number or exhibit low levels of atypia of the cells. Based on the results of the present study, we propose that Telo-CY is capable of addressing the challenges experienced by conv-CY in predicting PDAC metastasis, whether due to the viability, atypia, or small number of cancer cells.

Notably, 4 of 10 patients who were positive on Telo-CY alone did not exhibit relapse with peritoneal dissemination; all 4 of these individuals yielded false-positive results on Telo-CY assessment. Accordingly, false-positive Telo-CY results would argue against the use of this Telo-CY method. As described in “Materials and Methods”, for Telo-CY testing, v-PTCs were inferred for GFP+ CD45− samples and those that tested positive for any of the three tested cancer biomarkers (Ep-CAM, CEA, or CA19-9). Notably, of the 10 patients who tested positive on Telo-CY alone, all 6 of those that exhibited relapse with peritoneal recurrence also possessed v-PTCs that expressed Ep-CAM; 3 of the remaining 4 patients who tested positive on Telo-CY alone possessed v-PTCs that did not express this molecule, and they did not relapse with peritoneal dissemination. Moreover, the 2 patients who were double-CY+ possessed v-PTCs that expressed Ep-CAM, and both exhibited peritoneal recurrence. Therefore, we suggest that the false-positive rate obtained by Telo-CY may be decreased significantly by the additional inclusion of testing for Ep-CAM expression, a parameter that appears to be an essential marker of Telo-CY+ malignant cells (Table 3). Collectively, our results indicate that findings obtained by conv-CY and Telo-CY are complementary and can provide mutual support for each other’s limitations.

Recently, the number of resectable or borderline-resectable PDAC patients receiving preoperative therapies has been reported to be increasing^36–38^. The detection, by conv-CY, of cancer cells in peritoneal lavage fluid remains difficult, an observation that may be attributable to the small number of such cells, the existence of atypical cells, or issues with cell viability. Furthermore, the need for conversion surgery in PDAC patients considered unresectable should be evaluated after the use of strong, long-term, preoperative chemotherapies; in this context, the results of CY may lead to a more-accurate assessment of resectability status and the implementation of appropriate therapeutic strategies for such patients. Notably, Satoi et al. reported the efficacy, in PDAC patients that demonstrate peritoneal metastasis, of a regimen that includes intravenous and intraperitoneal dosing with paclitaxel in combination with S-1; a portion of the patients treated with this regimen subsequently underwent conversion surgery^58^. To select relevant therapeutic strategies, including the continuation/cessation of treatments and the potential use of conversion surgery, CY examinations with higher sensitivity will be required. In addition, the utility of cytological findings, as obtained both by conv-CY and Telo-CY in samples recovered by staging laparoscopy, should be further evaluated in the future, especially given that the present study included only patients who underwent open laparotomy. Therefore, we propose that preoperative combined CY (using both conv-CY and Telo-CY) should be performed for all patients with PDAC, despite associated increases in time and medical costs.

There were several limitations to this study. First, this study was conducted at a single institution and enrolled a small number of patients. Second, treatment strategies, including neoadjuvant or adjuvant therapies, were not consistent, reflecting the fact that the standard therapies for PDAC continued to change and underwent development during this study period. Therefore, our study did not consider the utility of CY status in the context of specific perioperative therapies. Nonetheless, we note that the use of high-quality CY examination, using Telo-CY, improved the predictive diagnostic sensitivity for detection of occult peritoneal tumor spread in patients with PDAC.

In conclusion, combined CY examinations using both conv-CY and Telo-CY may enhance the accuracy of prediction of peritoneal recurrence in patients with PDAC. We propose that the status of CY should be confirmed by staging laparoscopy prior to curative resection.

## Materials and methods

### Ethics statement

All patients provided written, informed consent for the use of peritoneal lavage fluid for both conventional cytology and for research cytology; the written consent was recorded in the patients’ electronic health records. The protocol for this study, which was conducted as a single-institution trial, was approved (approvals nos. 422 and 444) by the Institutional Review Boards of the Osaka Police Hospital. This study was conducted according to the principles of the Declaration of Helsinki.

### Patients

Between January 2015 and February 2019, this study enrolled patients who presented at the Osaka Police Hospital with resectable PDAC, as assessed by cytological or histological evaluation. All patients had a confirmed pathological diagnosis of PDAC. Patients who had detectable macroscopic liver metastases, para-aortic lymph node metastases, or peritoneal dissemination at open laparotomy were excluded. The present study included PDAC cases that underwent curative pancreatectomy with lavage cytology. Written, informed consent was obtained from all patients prior to any study procedures or treatments. Clinicopathological factors and clinical stage were classified using the criteria of the Union for International Cancer Control (UICC, 8th Ed.).

### Abdominal exploration and sample collection

Abdominal exploration and collection of peritoneal lavage fluid were performed by open laparotomy. Under general anesthesia, peritoneal lavage cytology was performed using standard methods at the beginning of the abdominal exploration. Specifically, lavage was conducted using 100 mL of sterile 0.9% sodium chloride, which was introduced into the recto-uterine pouch (pouch of Douglas), gently stirred, and then immediately aspirated from the pouch. The 100 mL of lavage fluid were divided into two samples of 50-mL each; 50 mL of fluid were examined for conv-CY, and 50 mL of the remaining fluid were analyzed for Telo-CY, as described later. Any metastatic findings were confirmed by visual examination and palpation, as assessed by the presence or absence of liver metastasis and of peritoneal dissemination. The final results of CY were obtained after the resection had been performed; surgical resection was performed irrespective of the status on CY.

### Surgery and perioperative therapy

Resectability was assessed by computed tomography and magnetic resonance imaging according to the National Comprehensive Cancer Network (NCCN) guidelines^15^. From 2015 to 2017, upfront surgery was performed for patients with resectable PDAC. Subsequently (since 2018), either neoadjuvant chemotherapy (NAC; consisting of the combination of gemcitabine and S-1) or neoadjuvant chemoradiotherapy [NACRT; consisting of the combination of gemcitabine and S-1 administered concurrently with 50 Gy of intensity-modulated radiation therapy (IMRT)] was administered to patients ≤ 80 years of age, as suggested by the results of Eguchi et al.^59^. Macroscopic peritoneal dissemination and liver metastasis were deemed contraindications for surgical resection. For adjuvant chemotherapy, S-1 was administered primarily based on the results of the Japanese Study Group of Adjuvant Therapy for Pancreatic Cancer (JASPAC)-01 study^6^.

### Peritoneal lavage cytology

For conventional CY (conv-CY), 50 mL of the peritoneal lavage fluid were subjected to pathological examination. Smears were prepared using centrifuged deposits, subjected to conventional Papanicolaou and/or Giemsa staining, and carefully evaluated by at least two experienced pathologists in our hospital’s Pathology Department. In terms of Papanicolaou classification, Class II (negative for malignancy) was deemed CY−, Class III (atypical) was CY±, and Classes IV (suspicious for malignancy) and V (positive for malignancy) were CY+^32^. To further assess the presence of cancer cells, MOC-31 (dilution 1:100; Cat No. MA5-12442, Invitrogen, Waltham, MA, USA) immunostaining was also performed. To obtain further information, conv-CY using 50 mL of sterile 0.9% sodium chloride was performed for the subhepatic space (Morison’s pouch) in a similar manner to that described above. For TelomeScan F35 CY (Telo-CY), 50 mL of the remaining lavage fluid were analyzed to detect viable peritoneal tumor cells (v-PTCs) using the TelomeScan F35 system as described previously^30,31,58^. Briefly, cells recovered from 50 mL of peritoneal lavage fluid were incubated at 37 °C for 24 h with 1 × 10^9^ viral particles (VP) of rAdF35-142 T-GFP (TelomeScan F35). Following this incubation, to distinguish cells from leucocytes, the cells were washed and stained with anti-human CD45 antibody^60^ (dilution 1:40; Clone HI30; BioLegend, San Diego, CA, USA) and then observed by fluorescence microscopy. Human v-PTCs and false-positive cells were defined as those that tested GFP + CD45− and GFP+ CD45+, respectively. To further distinguish cancer cells, cells were labeled with monoclonal anti-Ep-CAM (dilution 1:100; ab7504; Abcam, Cambridge, UK), anti-CEA (dilution 1:30; M7072, clone II-7; DAKO, San Diego, CA, USA), and anti-CA19-9 (dilution 1:20; ab116024, clone CA19-9-203; Abcam) antibodies. GFP+, CD45−, and Ep-CAM+, CEA+ and/or CA19-9+ cells were counted as v-PTCs. For both conv-CY and Telo-CY, CY+ was defined as the presence of cancer cells in the peritoneal lavage fluid.

### Statistical analysis

Clinicopathological data were compared between CY+ and CY− patients. Where relevant, data are presented as medians with interquartile range or as means ± standard deviation (SD). Data for CY status were compared using Pearson’s (uncorrected) χ^2^ test, Fisher’s exact test, and the Mann–Whitney *U* test for independence, as appropriate. Overall survival (OS) was defined as the time from the date of surgery to either the date of death or last follow-up. Survival curves were generated using Kaplan–Meier methods and compared using the log-rank-test. The level of significance was set at *P*-value < 0.05. Statistical analyses were performed using JMP Pro statistical software (version 15.0.0; SAS Institute, Inc., Cary, NC, USA).

### Supplementary Information

Supplementary Information.

## Data Availability

The datasets generated during and/or analyzed during the current study are available from the corresponding author on reasonable request.
