# Supplementary material for: Clinical impact of high-quality testing for peritoneal lavage cytology in pancreatic cancer
Source: Sci Rep. 2024 May 3;14:10199. doi: 10.1038/s41598-024-60936-4 (PMC11068862; doi:10.1038/s41598-024-60936-4)
Supplement: Supplementary file 1 — Supplementary Information. [file 41598_2024_60936_MOESM1_ESM.pdf]

Clinical impact of high-quality testing for peritoneal lavage cytology in pancreatic cancer

Masahiro Tanemura[1]*, Kenta Furukawa[2], Manabu Mikamori[2], Tadafumi Asaoka[2], Hironao Yasuoka[3], Daiki Marukawa[1], Yasuo Urata[4], Daisaku Yamada[5], Shogo Kobayashi[5], Hidetoshi Eguchi[5]

[1] Department of Surgery, Rinku General Medical Center, 2-23 Rinkuourai-kita, Izumisano, Osaka 598-8577, Japan

Departments of [2] Surgery and [3] Pathology, Osaka Police Hospital, 10-31 Kitayamachyo Tennoujiku, Osaka 543-0035, Japan

[4] Oncolys BioPharma Inc., Toranomon Towers 10F, 4-1-28 Toranomon Minato-ku, Tokyo 105-0001, Japan

[5] Department of Gastroenterological Surgery, Graduate School of Medicine and Faculty of Medicine, Osaka University, 2-2 Yamadaoka, Suita, Osaka 565-0871, Japan

*Correspondence and request for materials should be addressed to M.T. (e-mail: mtanemuram64@gmail.com)

Summary of the peritoneal recurrence rate in each group by combined cytological diagnosis

Supplemental Figure 1 shows the summary of the peritoneal recurrence rate in each cytological diagnostic group. Two patients tested double-CY+ and relapsed early with peritoneal recurrence after curative surgery (peritoneal recurrence rate=100%). For double-CY-negative patients, of the 38 patients who were conv-CY− and Telo-CY−, as well as conv-CY± patients, only 3 patients relapsed with peritoneal recurrence postoperatively (peritoneal recurrence rate=7.9%). The peritoneal recurrence rate of double-CY+ patients was significantly higher than that of double-CY− patients (P=0.023). Although 6 of 10 patients positive only on Telo-CY died following peritoneal recurrence, with deaths occurring 8-42 months after the curative resections (peritoneal recurrence rate=60%), no peritoneal recurrences were observed in 3 patients positive only on conv-CY (peritoneal recurrence rate=0%).

The peritoneal recurrence rate of the 12 patients with Telo-CY+ status, including 2 double-CY+ individuals and 10 positive on Telo-CY alone, is significantly higher than that of the 38 patients who were double-CY− [peritoneal recurrence rate, 67% (Telo-CY+) and 7.9% (double-CY−); P=0.034]. In contrast, no significant differences in peritoneal recurrence rates are observed between double-CY− patients (n=38) and conv-CY+ status, including 2 double-CY+ individuals and 3 positive on conv-CY alone [peritoneal recurrence rate, 7.9% (double-CY−) and 40%

(conv-CY+); P=0.244]. Collectively, these results suggest that PDAC patients with Telo-CY+ status may belong to the high-risk group for peritoneal recurrence.

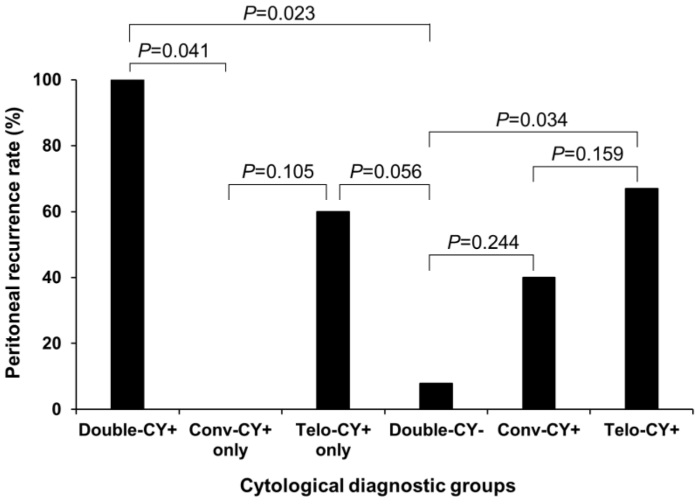

Supplemental Figure 1. Peritoneal recurrence rate in each cytological diagnostic group

CY, peritoneal lavage cytology; conv-CY, Papanicolaou-based conventional CY; Telo-CY, TelomeScan-based CY. Double-CY+ are patients positive on both conv-CY and Telo-CY (n=2). Conv-CY+ only are patients diagnosed as positive only on conv-CY (n=3). Telo-CY+ only are patients diagnosed as positive only on Telo-CY (n=10). Double-CY-negative patients are conv-CY− and Telo-CY−, as well as conv-CY± patients (n=38). Conv-CY-positive patients are positive only on conv-CY (n=3) and double-CY-positive (n=2). Telo-CY-positive patients are positive only on Telo-CY (n=10) and double-CY-positive (n=2). Data for peritoneal recurrence rates in each CY group are compared using Pearson's (uncorrected) χ^2 test.

Representative CY findings of a patient class III on conv-CY.

Supplemental figure 2 shows the cytological and immunocytochemical findings from a patient who was class III on conv-CY and Telo-CY negative. When assessed by Papanicolaou staining in conv-CY, cells from this individual displayed malignant findings, including a large nucleus/cytoplasm (N/C) ratio. However, these cells did not stain positive for MOC-31 (Supplemental Fig. 2). Accordingly, these cells were defined as class III based on the Papanicolaou classification system [32]. In contrast, Telo-CY of viable cells obtained from this patient was GFP+ and CD45-, and demonstrated no expression of all tumor markers on viable Telo-CY-positive cells, including EpCAM, CEA, and CA19-9 (Supplemental Fig. 2). Therefore, the viable cells recovered in peritoneal lavage fluid and detected by TelomeScan F35 were not judged to be viable cancer cells. For clinical findings after operation, this patient, who tested class III on conv-CY alone, did not relapse with peritoneal recurrence of PDAC and survived for 46 months postoperatively.

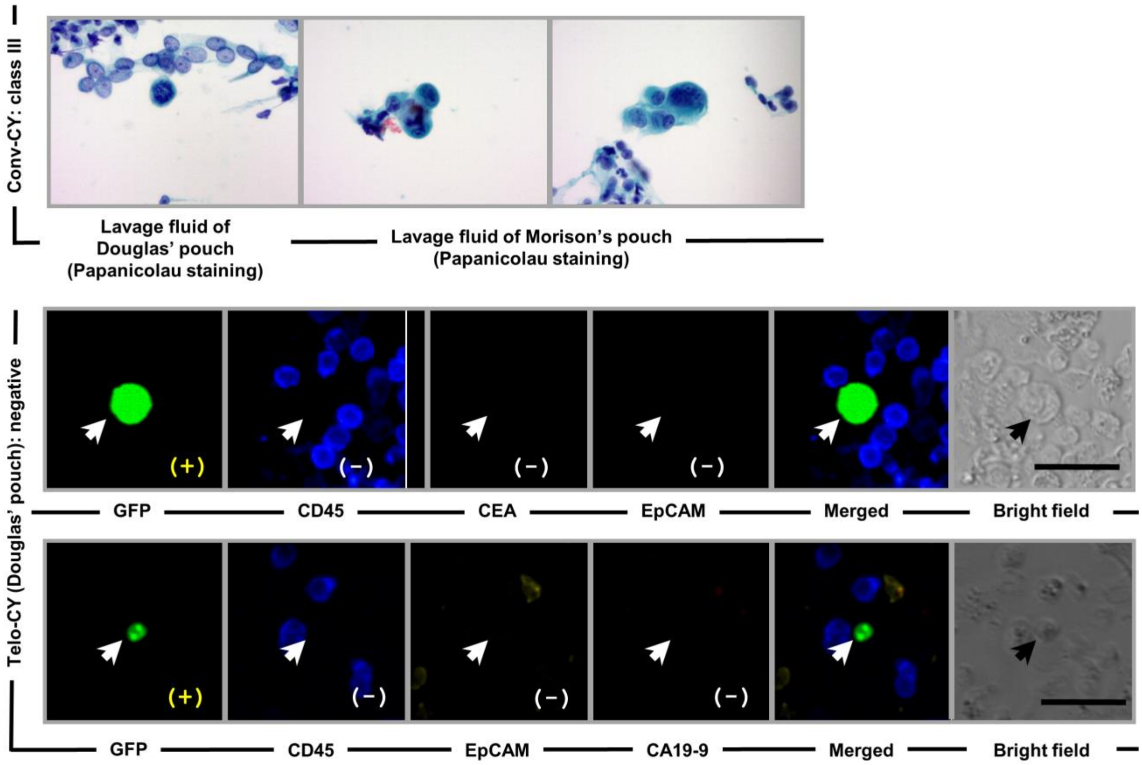

Supplemental Figure 2. Representative CY findings of a patient class III on conv-CY alone.

Top: conv-CY findings, with Papanicolaou staining.

Bottom: Telo-CY findings, including GFP fluorescence; immunocytochemical staining of CD45, CEA, or Ep-CAM; merged fluorescence; and bright-field image.

CY, peritoneal lavage cytology; conv-CY, Papanicolaou-based conventional CY; Telo-CY, TelomeScan-based CY; CEA, carcinoembryonic antigen; Ep-CAM, epithelial cell adhesion molecule (CD326); CD45 (i.e. leukocyte common antigen, protein tyrosine phosphatase type C: PTPRC); GFP, green fluorescence protein

White and black arrows show v-PTCs. Scale bar: 30 μm

Detailed results of peritoneal lavage cytology with conv-CY and Telo-CY.

Supplemental Table 1 shows the detailed results of conv-CY and Telo-CY. Two patients tested double-CY+, and another 3 patients tested positive only on conv-CY. In contrast, 10 patients were diagnosed positive only on Telo-CY. Two patients who tested conv-CY± (Class III) also tested Telo-CY−. The remaining 36 patients were double-CY−.

For Telo-CY diagnosis, the actual number of GFP+/CD45- cells, cell viability of cells recovered in peritoneal fluid and Telo-CY diagnosis are shown in Supplemental Table 1. No significant differences in both cell number of GFP+/CD45− and cell viability were observed in comparisons of the five groups: double-CY+, conv-CY+ only, Telo-CY+ only, conv-CY± (Class III) and double-CY−.

Supplemental Table 1. Detailed results of peritoneal lavage cytology with conv-CY and Telo-CY, including cell number and cell viability.

**Combined cytological diagnosis (n)**	**Conv-CY diagnosis: +/- (MOC-31 staining: +/-)**	**Telo-CY diagnosis: +/- (Cell number of v-PTCs)**	**Number of GFP+/CD45- cells**	**Cell viability (%)**
Double-CY+ (2)	Conv-CY: + (MOC-31: +)	Telo-CY: + (16)	215	73
	Conv-CY: + (MOC-31: +)	Telo-CY: + (6)	34	43
Conv-CY+ only (3)	Conv-CY: + (MOC-31: +)	Telo-CY: – (0)	1571	86
	Conv-CY: + (MOC-31: -) Large N/C ratio	Telo-CY: – (0)	37	71

	Conv-CY: +<br>(MOC-31: +)	Telo-CY: – (0)	6	95
Telo-CY+only(10)	Conv-CY: -<br>(MOC-31: -)	Telo-CY: + (100)	2887	40
	Conv-CY: -<br>(MOC-31: -)	Telo-CY: + (44)	1020	61
	Conv-CY: -<br>(MOC-31: -)	Telo-CY: + (2)	23	97
	Conv-CY: -<br>(MOC-31: -)	Telo-CY: + (2)	37	32
	Conv-CY: -<br>(MOC-31: -)	Telo-CY: + (9)	291	91
	Conv-CY: -<br>(MOC-31: -)	Telo-CY: + (6)	13	91
	Conv-CY: -<br>(MOC-31: -)	Telo-CY: + (28)	310	96
	Conv-CY: -<br>(MOC-31: -)	Telo-CY: + (39)	1886	97
	Conv-CY: -<br>(MOC-31: -)	Telo-CY: + (19)	571	36
	Conv-CY: -<br>(MOC-31: -)	Telo-CY: + (7)	816	44
Conv-CY±<br>(class III) (2)	Conv-CY: ±<br>(MOC-31: -)<br>Large N/C ratio	Telo-CY: – (0)	4	40
	Conv-CY: ±<br>(MOC-31: -)<br>Large N/C ratio	Telo-CY: – (0)	10	12
Double-CY- (36)	Conv-CY: -<br>(MOC-31: -)	Telo-CY: – (0)	461.3 (mean)	76.3<br>(mean)

v-PTCs, viable peritoneal tumor cells; CY, peritoneal lavage cytology; conv-CY, Papanicolaou-based conventional CY; Telo-CY, TelomeScan-based CY; N/C ratio, nucleus/cytoplasm ratio; MOC-31 staining, immunostaining by anti-human epithelial-related antigen (clone MOC-31) monoclonal Ab;

CD45 (i.e. leukocyte common antigen, protein tyrosine phosphatase type C: PTPRC); GFP, green fluorescence protein; N.D., not done.

Double-CY+ were patients positive on both conv-CY and Telo-CY (n=2). Conv-CY+ only were patients positive only on conv-CY (n=3). Telo-CY+ only were patients positive only on Telo-CY (n=10). Conv-CY± were patients having atypical cells (Class III) on conv-CY, but not Telo-CY− (n=2). Double-CY− were patients negative on both conv-CY and Telo-CY (n=36).
